# Phylogenetic Tree Analysis of the Cold-Hot Nature of Traditional Chinese Marine Medicine for Possible Anticancer Activity

**DOI:** 10.1155/2017/4365715

**Published:** 2017-01-12

**Authors:** Xianjun Fu, Xuxia Song, Xuebo Li, Kah Keng Wong, Jiaoyang Li, Fengcong Zhang, Changyun Wang, Zhenguo Wang

**Affiliations:** ^1^School of Information Management, Shandong University of Traditional Chinese Medicine, Jinan 250355, China; ^2^Department of Neurology, Qingdao Municipal Hospital, Qingdao 266011, China; ^3^Department of Immunology, School of Medical Sciences, Universiti Sains Malaysia, Health Campus, 16150 Kubang Kerian, Kelantan, Malaysia; ^4^Key Laboratory of Marine Drugs, Ministry of Education of China, School of Medicine and Pharmacy, Ocean University of China, Qingdao, Shandong Province 266003, China

## Abstract

Traditional Chinese Marine Medicine (TCMM) represents one of the medicinal resources for research and development of novel anticancer drugs. In this study, to investigate the presence of anticancer activity (AA) displayed by cold or hot nature of TCMM, we analyzed the association relationship and the distribution regularity of TCMMs with different nature (613 TCMMs originated from 1,091 species of marine organisms) via association rules mining and phylogenetic tree analysis. The screened association rules were collected from three taxonomy groups: (1) Bacteria superkingdom, Phaeophyceae class, Fucales order, Sargassaceae family, and* Sargassum* genus; (2) Viridiplantae kingdom, Streptophyta phylum, Malpighiales class, and Rhizophoraceae family; (3) Holothuroidea class, Aspidochirotida order, and* Holothuria* genus. Our analyses showed that TCMMs with closer taxonomic relationship were more likely to possess anticancer bioactivity. We found that the cluster pattern of marine organisms with reported AA tended to cluster with cold nature TCMMs. Moreover, TCMMs with salty-cold nature demonstrated properties for softening hard mass and removing stasis to treat cancers, and species within Metazoa or Viridiplantae kingdom of cold nature were more likely to contain AA properties. We propose that TCMMs from these marine groups may enable focused bioprospecting for discovery of novel anticancer drugs derived from marine bioresources.

## 1. Introduction

The nature of traditional Chinese medicines (TCMs) can be classified into three categories (cold, hot, and neutral) that represent the types of body reactions after the administration of specific TCM [1, 2]. The therapeutic effect of CMs depends mainly on the nature of the drugs as well as the processes they regulate to recover the balance between Yin and Yang in the human body [3, 4]. According to TCM, the rationale for the correct remedy selection is based upon a corresponding TCM syndrome (Zheng, 证, or pattern) [5]. A patient will present with a syndrome upon disruption of Yin-Yang balance, which may be caused either by external and/or internal pathological factors. This can be regarded as clinical phenotype, such as cold or hot syndrome [5, 6]. The standard therapeutic guideline used to treat cold or hot syndrome is to “cure cold syndrome by medication with hot nature” and to “cure hot syndrome by medication with cold nature” [5]. This therapeutic practice has been validated and developed over thousands of years, and most CMs have thus been labeled with different nature types as an outcome of this repeated clinical practice [1]. Recent literature shows that different biological effects conferred by a specific TCM could serve as the basis to discriminate cold and hot nature of TCMs [7, 8]. Thus, conversely, the cold-hot nature of a specific TCM could potentially serve as clues to its bioactivities including anticancer properties.

As a vital part of TCMs, Traditional Chinese Marine Medicine (TCMM) has been used to treat and prevent diseases for thousands of years, and it is based on a unique theoretical framework, diagnosis, and treatment [9]. TCMM appears to be effective in treating various diseases such as cancers, malaria, diabetes, cardiocerebrovascular diseases, immunodeficiency diseases, and senile dementia, and therefore has become an important medicinal resource for the research and development of new drugs [10].

Cancer poses serious threat to human health worldwide, and there have been efforts in screening for compounds possessing anticancer activity (AA) from TCMMs [17]. Marine organisms including TCMMs have evolved efficient and highly potent metabolites that exhibit strong biological activity at low concentrations to circumvent rapid dilution caused by their aqueous environment [18], and this confers a potential advantage over metabolites of terrestrial origin including TCMs originating from nonmarine sources. Moreover, TCMMs contain significant differences from TCMs of terrestrial origin including their bioactivity properties, cold and hot nature [10, 19, 20]. Marine organisms have been demonstrated to be promising source of novel antitumor compounds [18, 21] and several of the marine families of TCMMs have been explored and reportedly show anticancer potential [10, 22].

High-throughput screening for novel anticancer drugs are widely conducted; however it is costly and might yield chemical hits with low actual clinical efficacy and/or high toxicity [23]. It has been reported that distinct plant species yields potent bioactive compounds at higher rates than other plant species, and most drugs are derived from preexisting drug-productive families [24]. Clues to drug-productive species can be obtained from the species-distribution profiles of phylogenetic tree [19]. Almost 80% of the approved drugs and 67% of the clinical-trial drugs concentrated in 17 and 30 drug-prolific families, respectively, including Fabid and Malvid groups of the Rosidae subclass, the Lamiid and Campanulid groups of the Asterid subclass, and the Ranunculales order [24]. Eribulin mesylate is a structurally simplified synthetic analogue of halichondrin B used for the treatment of metastatic breast cancer, which is a natural product isolated from the marine sponge* Halichondria okadai,* which originates from a drug-productive family Halichondriidae [25–27]. Taken together, these instances provide the basis to screen for natural resources possessing AA activities through phylogenetic tree analysis.

Thus, in this study, in order to examine the phylogenetic tree and cold-hot nature of TCMM for identifying TCMMs with potential AA properties, association rules mining and phylogenetic tree construction methodologies were used to investigate the association relationship and distribution regularity of TCMMs with different nature possessing AA properties.

## 2. Materials and Methods

### 2.1. Datasets Preparation

The cold-hot nature categorization of 613 TCMMs related to 1,091 marine bioresources species were retrieved from the “Chinese Marine Materia Medica” [22]. Latin name and taxonomy data of the related bioresources were retrieved from the National Center for Biotechnology Information (NCBI) Taxonomy Database (https://www.ncbi.nlm.nih.gov/taxonomy) [28]. The 1,091 marine species were clustered into three groups: cold, hot, and neutral.

The anticancer bioactivity information of each marine bioresources species were retrieved from PubMed literature database by using the following retrieval formula.

“Latin name of each marine bioresource species” [All Fields] AND (anticancer [All Fields] OR antitumer [All Fields] OR antitumor [All Fields]).

Each species was labeled with presence or absence of anticancer bioactivity according to the retrieved results. All the results were independently checked by two researchers, F. X. and S. X.

### 2.2. Phylogenetic Tree Construction

The phylogenetic trees were generated by using the NCBI taxonomy-based automatic tree generator against known families in the Bacteria, Viridiplantae, and Metazoa kingdoms or superkingdoms [24, 29].

First, TAX ID of each marine bioresources species were retrieved from The NCBI Taxonomy System (https://www.ncbi.nlm.nih.gov/taxonomy) [28]. Then data of TAX ID were input to phylot web (http://phylot.biobyte.de/index.html) to construct phylogenetic tree, and the visualization of the tree was conducted in iTOL (version 3.3.2) and EvolView [19, 24, 30, 31]. Family or species names were labeled at branch ends. Reported anticancer bioactivity clusters and different nature were labeled or marked in the phylogenetic trees.

### 2.3. Association Rules Mining

The association relationship between reported anticancer bioactivity and taxonomy or nature types was mined by aRules package [32] based on the R platform to elucidate the association rules.

An association rule is an implication of the form *X*⇒*Y*, where *X* ⊂ *I*, *Y* ⊂ *I*, and *X*∩*Y* = *⌀*. The rule *X*⇒*Y* holds in the database *D* with confidence and support [33]. The support is a measure of the frequency of a rule, and the confidence is a measure of the strength of the relation between sets of items [34]. In this study, the cold-hot nature and taxonomy data of TCMMs were taken as *X*, while the AA of each TCMM was regarded to *Y*; the association rules whose confidence and support were larger than the set thresholds (50.00% for confidence and 0.5% for support) were chosen as strong association rules.

## 3. Results and Discussion

### 3.1. Cluster Pattern of Marine Organism from TCMMs with Reported AA

In this study, 613 TCMMs originated from 1,091 species of marine organisms were screened for potential AA properties (Table 1). Majority of the species (*n* = 870 of 1,091; 79.74%) were from Metazoa kingdom. Among the 1,091 species investigated, 194 species were reported to have AA with nearly half of them (*n* = 92; 47.42%) from the Metazoa kingdom. More than half of the interrogated species within the Viridiplantae (green plants) kingdom (*n* = 45 of 89; 50.56%) and Bacteria superkingdom (*n* = 6 of 9; 66.67%) demonstrated AAs. This implies that the marine species of TCMMs from Viridiplantae kingdom and Bacteria superkingdom are more likely to possess AA.

**Figure 1:**
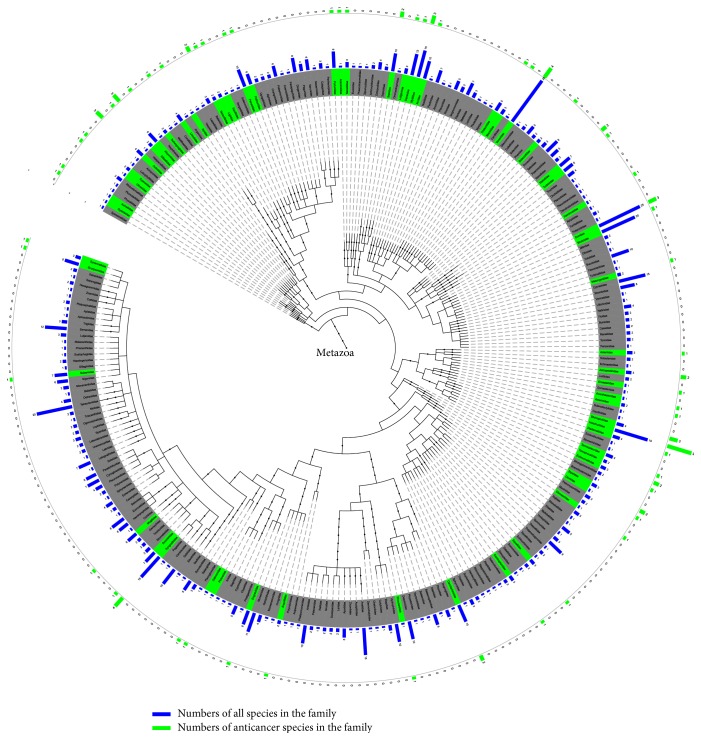
The distribution of marine anticancer activity families (green background color) phylogenetic tree of Metazoa kingdom. The family names are provided at branch ends, which can be viewed more clearly by enlarging the figure in the electronic version. The length of the blue and green bar outside the circle represents the number of all species and the AA species, respectively, in the family.

Metazoa kingdom showed 61 AA families concentrated in 38 clusters (Figure 1; Table S1 in Supplementary Material available online at https://doi.org/10.1155/2017/4365715). These families were present in seven of eight phylums known to possess AAs (Table S2). Four phylums contained more than ten AA species including 24 in Mollusca, 23 in Echinodermata, 22 in Chordata, and 10 in Cnidaria. There were 18 AA classes from 25 known classes of Metazoa (Table S3), and three classes (Holothuroidea, Actinopteri, and Bivalvia) contained more than ten AA species. Three orders (Aspidochirotida, Veneroida, and Alcyonacea) contained more than five AA species (Table S4).

This implies that TCMMs from Metazoa are potential candidates for anticancer drug discovery. Diverse peptides with a wide range of biological activities including antimicrobial and antitumoral have been isolated from different phyla of Mollusca, Cnidaria, and Echinodermata [35]. Two novel marine anticancer compounds, kahalalide F and ES285, have been isolated from the Indopacific mollusc* Elysia rufescens* and the North Atlantic mollusc* Spisula polynyma*, respectively [36]. The phylum Cnidaria is unique such that practically all of its members are toxic and contain Cnidarian toxins which are a rich source of polypeptides with a wide variety of biological activities including pore-forming cytolysins, phospholipases, neurotoxins, and protease inhibitors [35]. These marine organisms could be an important source of structurally bioactive secondary metabolites. There have been 12 reported novel and highly potent antitumor natural products derived from seven species of cnidarians of marine origin [37].

**Figure 2:**
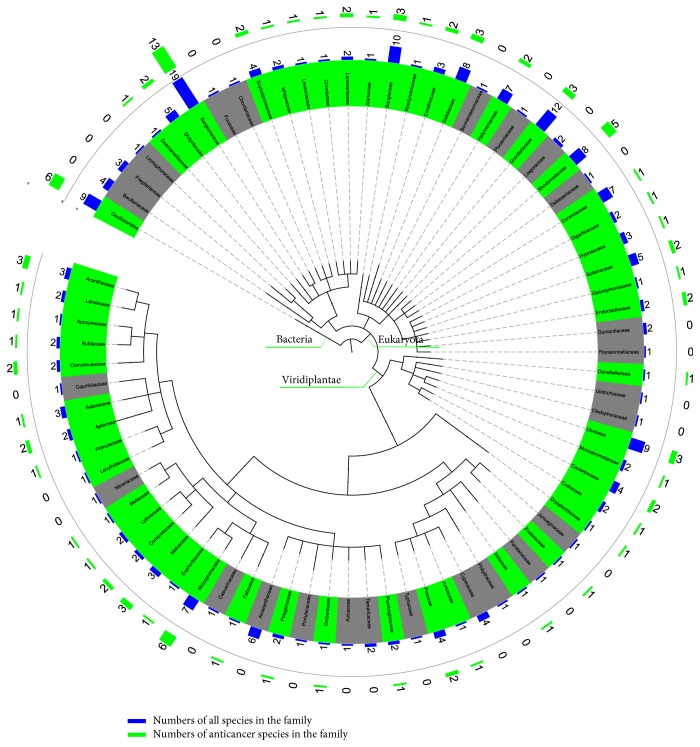
The distribution of marine families with AA in phylogenetic tree of Viridiplantae kingdom, Eukaryota superkingdom, and Bacteria superkingdom. Coloring and labeling schemes are as described in Figure 1.

Figure 2 presents the distribution of marine families with AA in phylogenetic tree of Viridiplantae kingdom, Eukaryota superkingdom, and Bacteria superkingdom. A total of 52 AA families were concentrated in 18 clusters (Figure 2; Table S5). These families were distributed in five AA phylums (Table S6) in which Streptophyta phylums of Viridiplantae contained 35 AA species. There were two classes from the Eukaryota superkingdom containing more than 20 AA species (Table S7), the Florideophyceae (24 AA species) and Phaeophyceae (23 AA species). One order (Fucales) contained more than ten AA species (Table S8).

Compared with Figure 1, Figure 2 showed more concentrated anticancer family clusters in Bacteria and Eukaryota superkingdom than Metazoa kingdom. Bacteria have widely contributed to some of the most useful chemotherapeutic drugs [38], while marine cyanobacteria contain antiproliferative properties, yielding several potent inhibitors of malignancies [39]. All of the six AA species of TCMMs from Bacteria superkingdom are of Cyanobacteria phylum.

Viridiplantae (green plants) are an ancient group of eukaryotes comprising of two main clades: the Chlorophyta and the Streptophyta. The former consists of a wide diversity of green algae while the latter consists of freshwater green algae and terrestrial plants [40]. There are four phyla of algae including red algae (Florideophyceae), brown algae (Phaeophyceae), green algae (Chlorophyta), and diatom (Bacillariophyceae) and two phyla of plants from coastal wetlands including Pteridophyta and Angiospermae [10]. Marine plants serve as main sources of potential anticancer agents [38].

### 3.2. Cold-Hot Nature Distribution of Marine Organism from TCMMs with Reported AA

Within the 1,091 marine organisms, 380 can be grouped into TCMMs with cold nature, 233 with hot nature, and 366 with neutral nature (Table 2). More than half of AA species were from the cold group (*n* = 51.03%), followed by the neutral (25.26%) and hot group (12.37%).

It was reported that basic pharmacological effects of herbals with cold nature are antibacterial, anti-inflammatory, antitumor, antipyretic, diuretic, lowering blood pressure, sedation, and analgesic [41]. Most frequently used TCMMs are generally of cold nature [20]. Studies have shown that salty flavor and cold nature (such as* Sargassum* and* Laminariae Thallus*) are representative of TCMMs [10, 20]. In terms of medicinal effects, the most representative efficacies of TCMMs with salty-cold flavor and nature (e.g.*, Sargassum*,* Laminariae Thallus*,* Ostreae Concha*, and* Meretricis Concha*) include softening hard mass and removing stasis to treat cancers [10]. This might serve as an explanation, at least partially, as to why AA species are often from the cold group.

### 3.3. The Association Rules and Phylogenetic Tree of Marine Organisms

The association rules mining resulted in 12 screened rules (Table 3). There were 11 rules with single item and one with double items. In the single item rules, one was of superkingdom (Bacteria with confidence of 66.67%) and one was associated with Streptophyta phylum, while two were related to Holothuroidea and Phaeophyceae classes. The Malpighiales order and Rhizophoraceae family showed strong association with AA with confidences of 87.50% and 85.71%, respectively. The double items of cold and Viridiplantae kingdom also showed strong association with AA, implying that the species of TCMMs with cold nature from Viridiplantae kingdom tend to have AA.

**Figure 3:**
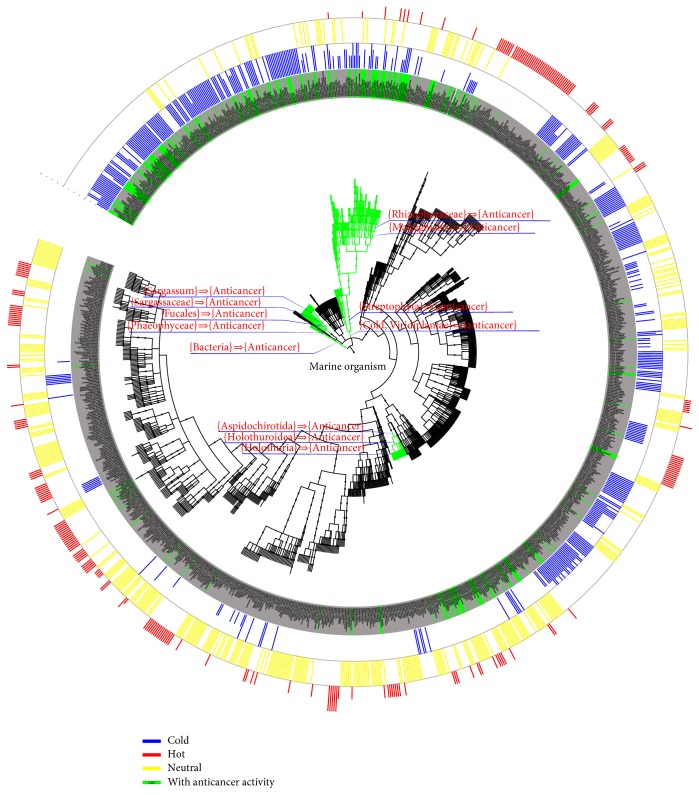
The distribution of marine AA species (green background) in the phylogenetic tree of marine organisms. The cold-hot nature of each species was labeled outside of the circle by different colors. The length of the bar represents the degree of nature of TCMMs. The screened association rules were labeled at the corresponding branch (the branches marked with different color). The names of species of the marine organisms of TCMMs are provided at branch ends, which can be viewed more clearly by enlarging the figure in the electronic version.

Figure 3 shows the cluster pattern of marine AA families, in the phylogenetic tree of marine organisms, tended to cluster with cold nature TCMMs. In contrast, few of the AAs-containing families clustered with hot and neutral nature TCMMs. The screened association rules labeled at the corresponding branch collected at three species groups (from superkingdom to genus) in the phylogenetic tree. The cluster pattern (Figure 3) contained three major groups: (1) the first group consisting of Bacteria superkingdom, Phaeophyceae class, Fucales order, Sargassaceae family, and* Sargassum* genus; (2) the second group consisting of Viridiplantae kingdom, Streptophyta phylum, Malpighiales class, and Rhizophoraceae family; (3) the third group comprising three levels of Holothuroidea class, Aspidochirotida order, and* Holothuria* genus at the same branch.

The results above suggest that certain species of TCMMs with closer taxonomic relationship are more likely to have AA. The AA of marine organisms is mostly based on the secondary metabolites of each species [42, 43]. The distribution of secondary metabolites has some value for taxonomy [44]. Chemical structure of secondary metabolites forms the molecular basis for its bioactivity [45], and marine natural products are important sources of chemical scaffolds [46]. Natural products from marine species with closer taxonomic relationship contain similar scaffolds and bioactivities [47]. For instance, the marine organisms* Sargassum fusiforme*,* Sargassum hemiphyllum*,* Sargassum pallidum*,* Sargassum carpophyllum*,* Sargassum horneri*, and* Sargassum thunbergii* are from the Sargassaceae family that form the TCMM seaweed (known as “haizao” in Chinese or 海藻) with cold nature. All of the* Sargassum *seaweed possessed phytosterols compounds with the same scaffold (compounds 1–5 in Table 4) or similar structure (compounds 6 in Table 4) containing similar anticancer bioactivity.

In addition to the grouped species of TCMMs with closer taxonomic relationship, TCMM species with cold nature from Viridiplantae kingdom also showed a tendency for AA with confidence of 56%. As discussed above, marine plants contain potential anticancer agents and cold nature TCMMs render softening of hard mass and stasis removal. Hence, the species (e.g.,* Ulva pertusa* [48]) with combination of Viridiplantae kingdom and cold nature are more likely to demonstrate AA. For example,* Ulva pertusa* from the Ulvaceae family of Viridiplantae kingdom was used as classic TCMM with cold nature to treat thyroid neoplasm from Tang dynasty and recorded in the herbal book of “Bencao Shiyi (Supplement to Materia Medica, 本*草拾遗*)” [22]. It is reported that* Ulva pertusa* showed antitumor activity against Meth-A fibrosarcoma by intraperitoneal administration of 50 mg/kg daily for seven days [49].

Nonetheless, we acknowledge limitations of the study as follows: (1) the studies included in the phylogenetic tree analysis took into account results from in vitro investigations. However, in vitro studies have remained the prerequisite before a candidate compound is tested further in in vivo or human trials settings, and excluding results from in vitro studies could significantly reduce the sensitivity of our analysis; (2) this study represented TCMMs originated from species of marine organisms for potential AA properties available currently and several more are being actively discovered.

Our previous study showed that the TCMM from the organisms in the same family may have the same nature, while marine plants such as Chlorophyta, Florideophyceae, and Phaeophyceae were associated with cold nature, and marine animals including Decapoda, Malacostraca, and Arthropoda contained close relationship with hot nature [19]. The different nature types seem to affect different biological processes based on the pluralistic character of molecular structure [50]. For distribution of secondary metabolites from marine species with closer taxonomic relationship, they contain similar scaffolds and bioactivities [47]. Marine algae associated with cold nature such as Chlorophyta, Florideophyceae, and Phaeophyceae contain antitumor properties [51].

## 4. Conclusions

Our analysis demonstrated that potential AA derived from Metazoa or Viridiplantae species with cold nature tended to have close taxonomic relationship than distantly distributed in phylogenetic tree. The clustered patterns with mined association rules presented in this work provide information pertaining to the groups of species with anticancer properties. Moreover, we have shown that phylogenetic tree analysis can be utilized to shortlist plant or animal species that possess potential AA. Future bioprospecting studies on TCMMs are thus warranted with aims of producing novel anticancer drugs.

## Supplementary Material

Supplementary Material contains tables of the distribution of marine organisms with anticancer activity (AA) in different families, phylums, classes, orders of Metazoa and Viridiplantae kingdom, Eukaryota superkingdom and Bacteria superkingdom, in which this distribution was annotated with Percentage derived from division with the total number of species.

## Figures and Tables

**Table 1:** The distribution of marine species with AA in different kingdom or superkingdom.

Kingdom or Superkingdom^#^	Number of species (%)^a^	Number of species with AA (%)^b^
Metazoa	870 (79.74%)	92 (10.57%)
Viridiplantae	89 (8.16%)	45 (50.56%)
Others in Eukaryota^*#*^	123 (11.27%)	51 (41.46%)
Bacteria^*#*^	9 (0.82%)	6 (66.67%)
Total	1,091 (100%)	194 (17.78%)

^a^Percentage derived from division with the total number of species within all kingdom and superkingdom combined (*n* = 1,091).

^b^Percentage derived from division with the number of species within each corresponding kingdom or superkingdom.

# refers to superkingdom.

**Table 2:** The distribution of marine organisms with AA in different nature categories.

Nature	Number of species (%)^a^	Number of species with AA (%)^b^
Cold	380 (34.83)	99 (26.05)
Hot	233 (21.36)	24 (10.30)
Neutral	366 (33.55)	49 (13.39)
None	112 (10.27)	22 (19.64)
Total	1,091 (100.00)	194 (17.78)

^a^Percentage derived from division with the total number of species within all different natures combined (*n* = 1,091).

^b^Percentage derived from division with the number of species within each corresponding nature.

**Table 3:** The results of association rules mining (support > 0.5%, confidence > 50%, lift > 2).

Rule ID	Single or double items	Taxonomic rank	Rules	Support	Confidence	Lift
1	Single item	Super kingdom	{*Bacteria*}⇒{anticancer}	0.55%	66.67%	3.75

2	Single item	Phylum	{*Streptophyta*}⇒{anticancer}	3.21%	52.24%	2.94

3	Single item	Class	{*Phaeophyceae*}⇒{anticancer}	2.20%	63.16%	3.55
4			{*Holothuroidea*}⇒{anticancer}	1.28%	51.85%	2.92

5	Single item	Order	{*Malpighiales*}⇒{anticancer}	0.64%	87.50%	4.92
6			{*Fucales*}⇒{anticancer}	1.19%	65.00%	3.66
7			{*Aspidochirotida*}⇒{anticancer}	1.10%	52.17%	2.93

8	Single item	Family	{*Rhizophoraceae*}⇒{anticancer}	0.55%	85.71%	4.82
9			{*Sargassaceae*}⇒{anticancer}	1.19%	68.42%	3.85

10	Single item	Genus	{*Sargassum*}⇒{anticancer}	1.01%	68.75%	3.87
11			{*Holothuria*}⇒{anticancer}	0.73%	61.54%	3.46

12	Double items	Nature & kingdom	{cold, *Viridiplantae*}⇒{anticancer}	2.57%	56.00%	3.15

**Table 4:** Anticancer compounds from species of Sargassaceae family of the TCMM Seaweed (Haizao, 海藻).

ID	Name	Structure		Species	AA
		Scaffold	R		
1	Fucosterol [11–16]	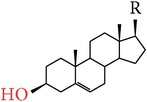	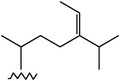	(1) *Sargassum fusiforme* (2) *Sargassum hemiphyllum* (3) *Sargassum pallidum* (4) *Sargassum carpophyllum* (5) *Sargassum horneri* (6) *Sargassum thunbergii*	(1) Induce the deformation activity of rice blast fungus (2) Inhibit the growth of P-388, T47D, and HT29 cell lines.

2	Sargasterol [13, 14]	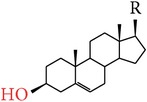	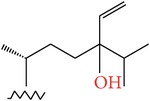	(1) *Sargassum fusiforme* (2) *Sargassum hemiphyllum* (3) *Sargassum pallidum* (4) *Sargassum carpophyllum*	Induce the deformation activity of rice blast fungus

3	24- Hydrogen peroxide based -24- vinyl cholesterol [13]	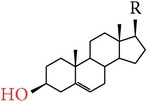	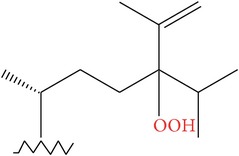	(1) *Sargassum fusiforme* (2) *Sargassum hemiphyllum* (3) *Sargassum carpophyllum*	(1) Induce the deformation activity of rice blast fungus (2) Inhibit the growth of HL-60 cell line.

4	24S, 28S- epoxy -24- ethyl cholesterol [13]	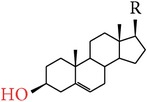	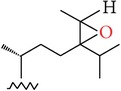	(1) *Sargassum fusiforme* (2) *Sargassum carpophyllum*	(1) Induce the deformation activity of rice blast fungus (2) Inhibit the growth of MCF-7, HCT-8, 1A9, HOS, and PC-3 cell lines.

5	Cholesterol -24- ketone [13]	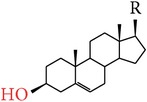	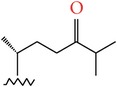	*Sargassum carpophyllum*	Induce the deformation activity of rice blast fungus

6	24-Ethylcholest-4-en-3,6-dione [13]	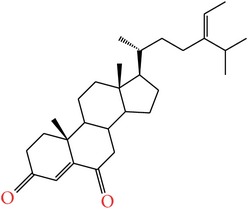		*Sargassum carpophyllum*	(1) Induce the deformation activity of rice blast fungus (2) Inhibit the growth of P-388 cell line.
