# Supplementary material for: Phylogenetic Tree Analysis of the Cold-Hot Nature of Traditional Chinese Marine Medicine for Possible Anticancer Activity
Source: Evid Based Complement Alternat Med. 2017 Jan 12;2017:4365715. doi: 10.1155/2017/4365715 (PMC5278566; doi:10.1155/2017/4365715)
Supplement: Supplementary file 1 — Supplementary Material contains tables of the distribution of marine organisms with anticancer activity (AA) in different families, phylums, classes, orders of Metazoa and Viridiplantae kingdom, Eukaryota superkingdom and Bacteria superkingdom, in which this distribution was annotated with Percentage derived from division with the total number of species. [file 4365715.f1.doc]

**Phylogenetic Tree Analysis of the Cold-hot Nature of Traditional Chinese Marine Medicine for Anticancer Activity**

**Xianjun FU ^1*^, Xuxia SONG ^2^, Xuebo LI ^1^, Kah Keng WONG^3^, Jiaoyang LI ^1^, Fengcong ZHANG, Changyun WANG ^4^, and Zhenguo WANG^1*^**

*1. School of Information Management, Shandong University of Traditional Chinese Medicine, Jinan 250355, China*

*2. Department of Neurology, Qingdao Municipal Hospital, Qingdao, China 266011*

*3. Department of Immunology, School of Medical Sciences, Health Campus, Universiti Sains Malaysia, 16150 Kubang Kerian, Kelantan, Malaysia*

*4. Key Laboratory of Marine Drugs, Ministry of Education of China, School of Medicine and Pharmacy, Ocean University of China, Qingdao, Shandong Province 266003, China*

**Corresponding authors**

Xianjun Fu ([xianxiu@hotmail.com](mailto:xianxiu@hotmail.com)) and Zhenguo Wang ([zhenguow@126.com](mailto:zhenguow@126.com))

**Email addresses:**

Xuxia Song: songxx_1975@163.com

Xuebo Li: 33399675@qq.com

Kah Keng Wong: kahkeng@usm.my

Jiaoyang Li: 371652188@qq.com

Fengcong Zhang: 13608922688@126.com

Changyun Wang: changyun@ouc.edu.cn

**Table S1.** The distribution of marine organisms with anticancer activity (AA) in different families of *Metazoa* kingdom

| **No** | **Family** | **Number** | **Percentage^a^** | **No. of AA** | **Percentage^b^** | **Percentage^c^** |
| --- | --- | --- | --- | --- | --- | --- |
|  | *Holothuriidae* | 19 | 2.18 | 9 | 9.78 | 47.37 |
|  | *Syngnathidae* | 15 | 1.72 | 4 | 4.35 | 26.67 |
|  | *Veneridae* | 28 | 3.22 | 4 | 4.35 | 14.29 |
|  | *Actiniidae* | 4 | 0.46 | 3 | 3.26 | 75.00 |
|  | *Alcyoniidae* | 6 | 0.69 | 3 | 3.26 | 50.00 |
|  | *Conidae* | 25 | 2.87 | 3 | 3.26 | 12.00 |
|  | *Mytilidae* | 15 | 1.72 | 3 | 3.26 | 20.00 |
|  | *Stichopodidae* | 4 | 0.46 | 3 | 3.26 | 75.00 |
|  | *Astropectinidae* | 3 | 0.34 | 2 | 2.17 | 66.67 |
|  | *Carcharhinidae* | 10 | 1.15 | 2 | 2.17 | 20.00 |
|  | *Haliotidae* | 7 | 0.80 | 2 | 2.17 | 28.57 |
|  | *Sepiidae* | 10 | 1.15 | 2 | 2.17 | 20.00 |
|  | *Styelidae* | 3 | 0.34 | 2 | 2.17 | 66.67 |
|  | *Tetraclitidae* | 2 | 0.23 | 2 | 2.17 | 100.00 |
|  | *Toxopneustidae* | 2 | 0.23 | 2 | 2.17 | 100.00 |
|  | *Acanthasteridae* | 1 | 0.11 | 1 | 1.09 | 100.00 |
|  | *Anguillidae* | 2 | 0.23 | 1 | 1.09 | 50.00 |
|  | *Apidae* | 1 | 0.11 | 1 | 1.09 | 100.00 |
|  | *Arcidae* | 10 | 1.15 | 1 | 1.09 | 10.00 |
|  | *Arenicolidae* | 1 | 0.11 | 1 | 1.09 | 100.00 |
|  | *Asteriidae* | 3 | 0.34 | 1 | 1.09 | 33.33 |
|  | *Asterinidae* | 2 | 0.23 | 1 | 1.09 | 50.00 |
|  | *Axinellidae* | 1 | 0.11 | 1 | 1.09 | 100.00 |
|  | *Centrophoridae* | 3 | 0.34 | 1 | 1.09 | 33.33 |
|  | *Chimaeridae* | 1 | 0.11 | 1 | 1.09 | 100.00 |
|  | *Cionidae* | 1 | 0.11 | 1 | 1.09 | 100.00 |
|  | *Cyaneidae* | 2 | 0.23 | 1 | 1.09 | 50.00 |
|  | *Diadematidae* | 4 | 0.46 | 1 | 1.09 | 25.00 |
|  | *Dysideidae* | 1 | 0.11 | 1 | 1.09 | 100.00 |
|  | *Echinometridae* | 3 | 0.34 | 1 | 1.09 | 33.33 |
|  | *Ellisellidae* | 2 | 0.23 | 1 | 1.09 | 50.00 |
|  | *Engraulidae* | 8 | 0.92 | 1 | 1.09 | 12.50 |
|  | *Haminoeidae* | 1 | 0.11 | 1 | 1.09 | 100.00 |
|  | *Ligiidae* | 2 | 0.23 | 1 | 1.09 | 50.00 |
|  | *Limulidae* | 4 | 0.46 | 1 | 1.09 | 25.00 |
|  | *Loliginidae* | 3 | 0.34 | 1 | 1.09 | 33.33 |
|  | *Mactridae* | 3 | 0.34 | 1 | 1.09 | 33.33 |
|  | *Melongenidae* | 2 | 0.23 | 1 | 1.09 | 50.00 |
|  | *Mugilidae* | 6 | 0.69 | 1 | 1.09 | 16.67 |
|  | *Muricidae* | 20 | 2.30 | 1 | 1.09 | 5.00 |
|  | *Nereididae* | 3 | 0.34 | 1 | 1.09 | 33.33 |
|  | *Oreasteridae* | 1 | 0.11 | 1 | 1.09 | 100.00 |
|  | *Ostreidae* | 12 | 1.38 | 1 | 1.09 | 8.33 |
|  | *Palinuridae* | 6 | 0.69 | 1 | 1.09 | 16.67 |
|  | *Penaeidae* | 10 | 1.15 | 1 | 1.09 | 10.00 |
|  | *Phocoenidae* | 1 | 0.11 | 1 | 1.09 | 100.00 |
|  | *Plotosidae* | 1 | 0.11 | 1 | 1.09 | 100.00 |
|  | *Portunidae* | 9 | 1.03 | 1 | 1.09 | 11.11 |
|  | *Salmonidae* | 1 | 0.11 | 1 | 1.09 | 100.00 |
|  | *Sciaenidae* | 7 | 0.80 | 1 | 1.09 | 14.29 |
|  | *Scombridae* | 7 | 0.80 | 1 | 1.09 | 14.29 |
|  | *Scorpaenidae* | 8 | 0.92 | 1 | 1.09 | 12.50 |
|  | *Solecurtidae* | 2 | 0.23 | 1 | 1.09 | 50.00 |
|  | *Squalidae* | 3 | 0.34 | 1 | 1.09 | 33.33 |
|  | *Squillidae* | 1 | 0.11 | 1 | 1.09 | 100.00 |
|  | *Subergorgiidae* | 2 | 0.23 | 1 | 1.09 | 50.00 |
|  | *Synanceiidae* | 3 | 0.34 | 1 | 1.09 | 33.33 |
|  | *Temnopleuridae* | 4 | 0.46 | 1 | 1.09 | 25.00 |
|  | *Tridacnidae* | 5 | 0.57 | 1 | 1.09 | 20.00 |
|  | *Turbinidae* | 6 | 0.69 | 1 | 1.09 | 16.67 |
|  | *Ulmaridae* | 1 | 0.11 | 1 | 1.09 | 100.00 |
|  | Others | 540 | 62.07 | 0 | 0.00 | 0.00 |
|  | Total | 870 | 100.00 | 92 | 100.00 | 10.57 |

a Percentage derived from division with the total number of species within *Metazoa* kingdom (n=870)

b Percentage derived from division with the number of species with AA within *Metazoa* kingdom (n=92).

c Percentage derived from division with the number of species within each corresponding family.

**Table S2.** The distribution of marine organisms with anticancer activity (AA) in different phylum of *Metazoa* kingdom

| **No** | **Phylum** | **Number** | **Percentage^a^** | **No. of AA** | **Percentage^b^** | **Percentage^c^** |
| --- | --- | --- | --- | --- | --- | --- |
|  | *Mollusca* | 282 | 32.41 | 24 | 26.09 | 8.51 |
|  | *Echinodermata* | 58 | 6.67 | 23 | 25.00 | 39.66 |
|  | *Chordata* | 407 | 46.78 | 22 | 23.91 | 5.41 |
|  | *Cnidaria* | 28 | 3.22 | 10 | 10.87 | 35.71 |
|  | *Arthropoda* | 84 | 9.66 | 9 | 9.78 | 10.71 |
|  | *Annelida* | 7 | 0.80 | 2 | 2.17 | 28.57 |
|  | *Porifera* | 3 | 0.34 | 2 | 2.17 | 66.67 |
|  | *Anthozoa* | 1 | 0.11 | 0 | 0.00 | 0.00 |
|  | Total | 870 | 100.00 | 92 | 100.00 | 10.57 |

a Percentage derived from division with the total number of species within *Metazoa* kingdom (n=870)

b Percentage derived from division with the number of species with AA within *Metazoa* kingdom (n=92).

c Percentage derived from division with the number of species within each corresponding phylum.

**Table S3.** The distribution of marine organisms with anticancer activity (AA) in different classes of *Metazoa* kingdom

| **No.** | **Class** | | **Number** | **Percentage^a^** | | **No. of AA** | | **Percentage^b^** | | **Percentage^c^** | |
| --- | --- | --- | --- | --- | --- | --- | --- | --- | --- | --- | --- |
|  | | *Holothuroidea* | 27 | | 3.10 | | 14 | | 15.22 | | 51.85 |
|  | | *Actinopteri* | 253 | | 29.08 | | 13 | | 14.13 | | 5.14 |
|  | | *Bivalvia* | 129 | | 14.83 | | 12 | | 13.04 | | 9.30 |
|  | | *Gastropoda* | 131 | | 15.06 | | 9 | | 9.78 | | 6.87 |
|  | | *Anthozoa* | 20 | | 2.30 | | 8 | | 8.70 | | 40.00 |
|  | | *Asteroidea* | 15 | | 1.72 | | 5 | | 5.43 | | 33.33 |
|  | | *Chondrichthyes* | 60 | | 6.90 | | 5 | | 5.43 | | 8.33 |
|  | | *Malacostraca* | 71 | | 8.16 | | 5 | | 5.43 | | 7.04 |
|  | | *Echinoidea* | 16 | | 1.84 | | 4 | | 4.35 | | 25.00 |
|  | | *Ascidiacea* | 4 | | 0.46 | | 3 | | 3.26 | | 75.00 |
|  | | *Cephalopoda* | 20 | | 2.30 | | 3 | | 3.26 | | 15.00 |
|  | | *Demospongiae* | 3 | | 0.34 | | 2 | | 2.17 | | 66.67 |
|  | | *Maxillopoda* | 6 | | 0.69 | | 2 | | 2.17 | | 33.33 |
|  | | *Polychaeta* | 7 | | 0.80 | | 2 | | 2.17 | | 28.57 |
|  | | *Scyphozoa* | 6 | | 0.69 | | 2 | | 2.17 | | 33.33 |
|  | | *Insecta* | 2 | | 0.23 | | 1 | | 1.09 | | 50.00 |
|  | | *Mammalia* | 26 | | 2.99 | | 1 | | 1.09 | | 3.85 |
|  | | *Merostomata* | 4 | | 0.46 | | 1 | | 1.09 | | 25.00 |
|  | | *Aves* | 42 | | 4.83 | | 0 | | 0.00 | | 0.00 |
|  | | *Hydrozoa* | 2 | | 0.23 | | 0 | | 0.00 | | 0.00 |
|  | | *Polyplacophora* | 2 | | 0.23 | | 0 | | 0.00 | | 0.00 |
|  | | *Reptilia* | 16 | | 1.84 | | 0 | | 0.00 | | 0.00 |
|  | | *Actiniaria* | 1 | | 0.11 | | 0 | | 0.00 | | 0.00 |
|  | | *Arachnida* | 1 | | 0.11 | | 0 | | 0.00 | | 0.00 |
|  | | Others | 6 | | 0.69 | | 0 | | 0.00 | | 0.00 |
|  | | Total | 870 | | 100.00 | | 92 | | 100.00 | | 10.57 |

a Percentage derived from division with the total number of species within *Metazoa* kingdom (n=870)

b Percentage derived from division with the number of species with AA within *Metazoa* kingdom (n=92).

c Percentage derived from division with the number of species within each corresponding class.

**Table S4.** The distribution of marine organisms with anticancer activity (AA) in different order of *Metazoa* kingdom

| **No.** | **Order** | **Number** | **Percentage^a^** | **No. of AA** | **Percentage^b^** | **Percentage^c^** |
| --- | --- | --- | --- | --- | --- | --- |
|  | *Aspidochirotida* | 23 | 2.64 | 12 | 13.04 | 52.17 |
|  | *Veneroida* | 61 | 7.01 | 7 | 7.61 | 11.48 |
|  | *Alcyonacea* | 12 | 1.38 | 5 | 5.43 | 41.67 |
|  | *Syngnathiformes* | 17 | 1.95 | 4 | 4.35 | 23.53 |
|  | *Actiniaria* | 4 | 0.46 | 3 | 3.26 | 75.00 |
|  | *Decapoda* | 68 | 7.82 | 3 | 3.26 | 4.41 |
|  | *Mytiloida* | 15 | 1.72 | 3 | 3.26 | 20.00 |
|  | *Temnopleuroida* | 6 | 0.69 | 3 | 3.26 | 50.00 |
|  | *Valvatida* | 5 | 0.57 | 3 | 3.26 | 60.00 |
|  | *Carcharhiniformes* | 16 | 1.84 | 2 | 2.17 | 12.50 |
|  | *Paxillosida* | 5 | 0.57 | 2 | 2.17 | 40.00 |
|  | *Perciformes* | 48 | 5.52 | 2 | 2.17 | 4.17 |
|  | *Semaeostomeae* | 3 | 0.34 | 2 | 2.17 | 66.67 |
|  | *Sessilia* | 5 | 0.57 | 2 | 2.17 | 40.00 |
|  | *Squaliformes* | 10 | 1.15 | 2 | 2.17 | 20.00 |
|  | *Stolidobranchia* | 3 | 0.34 | 2 | 2.17 | 66.67 |
|  | *Anguilliformes* | 19 | 2.18 | 1 | 1.09 | 5.26 |
|  | *Arcoida* | 11 | 1.26 | 1 | 1.09 | 9.09 |
|  | *Capitellida* | 1 | 0.11 | 1 | 1.09 | 100.00 |
|  | *Cetacea* | 20 | 2.30 | 1 | 1.09 | 5.00 |
|  | *Chimaeriformes* | 1 | 0.11 | 1 | 1.09 | 100.00 |
|  | *Clupeiformes* | 14 | 1.61 | 1 | 1.09 | 7.14 |
|  | *Diadematoida* | 4 | 0.46 | 1 | 1.09 | 25.00 |
|  | *Dictyoceratida* | 1 | 0.11 | 1 | 1.09 | 100.00 |
|  | *Echinoida* | 5 | 0.57 | 1 | 1.09 | 20.00 |
|  | *Enterogona* | 1 | 0.11 | 1 | 1.09 | 100.00 |
|  | *Forcipulatida* | 3 | 0.34 | 1 | 1.09 | 33.33 |
|  | *Halichondrida* | 1 | 0.11 | 1 | 1.09 | 100.00 |
|  | *Hymenoptera* | 2 | 0.23 | 1 | 1.09 | 50.00 |
|  | *Isopoda* | 2 | 0.23 | 1 | 1.09 | 50.00 |
|  | *Mugiliformes* | 6 | 0.69 | 1 | 1.09 | 16.67 |
|  | *Ostreoida* | 12 | 1.38 | 1 | 1.09 | 8.33 |
|  | *Phyllodocida* | 3 | 0.34 | 1 | 1.09 | 33.33 |
|  | *Salmoniformes* | 1 | 0.11 | 1 | 1.09 | 100.00 |
|  | *Scombriformes* | 14 | 1.61 | 1 | 1.09 | 7.14 |
|  | *Sepiida* | 10 | 1.15 | 1 | 1.09 | 10.00 |
|  | *Siluriformes* | 3 | 0.34 | 1 | 1.09 | 33.33 |
|  | *Teuthida* | 4 | 0.46 | 1 | 1.09 | 25.00 |
|  | *Xiphosura* | 4 | 0.46 | 1 | 1.09 | 25.00 |
|  | Others | 211 | 24.25 | 12 | 13.04 | 5.69 |
|  | Total | 870 | 100.00 | 92 | 100.00 | 10.57 |

a Percentage derived from division with the total number of species within *Metazoa* kingdom (n=870)

b Percentage derived from division with the number of species with AA within *Metazoa* kingdom (n=92).

c Percentage derived from division with the number of species within each corresponding order.

**Table S5.** The distribution of marine organisms with anticancer activity (AA) in different family of *Viridiplantae* kingdom, *Eukaryota* superkingdom and *Bacteria* superkingdom.

| **No.** | **Family** | **Number** | **Percentage^a^** | **Percentage^b^** | **No. of AA** | **Percentage^c^** |
| --- | --- | --- | --- | --- | --- | --- |
|  | *Sargassaceae* | 19 | 8.60 | 13 | 12.75 | 68.42 |
|  | *Oscillatoriales* | 9 | 4.07 | 6 | 5.88 | 66.67 |
|  | *Rhizophoraceae* | 7 | 3.17 | 6 | 5.88 | 85.71 |
|  | *Rhodomelaceae* | 8 | 3.62 | 5 | 4.90 | 62.50 |
|  | *Acanthaceae* | 3 | 1.36 | 3 | 2.94 | 100.00 |
|  | *Bangiaceae* | 10 | 4.52 | 3 | 2.94 | 30.00 |
|  | *Gelidiaceae* | 8 | 3.62 | 3 | 2.94 | 37.50 |
|  | *Gracilariaceae* | 12 | 5.43 | 3 | 2.94 | 25.00 |
|  | *Malvaceae* | 3 | 1.36 | 3 | 2.94 | 100.00 |
|  | *Ulvaceae* | 9 | 4.07 | 3 | 2.94 | 33.33 |
|  | *Apiaceae* | 2 | 0.90 | 2 | 1.96 | 100.00 |
|  | *Caulerpaceae* | 4 | 1.81 | 2 | 1.96 | 50.00 |
|  | *Combretaceae* | 2 | 0.90 | 2 | 1.96 | 100.00 |
|  | *Convolvulaceae* | 2 | 0.90 | 2 | 1.96 | 100.00 |
|  | *Corallinaceae* | 3 | 1.36 | 2 | 1.96 | 66.67 |
|  | *Dictyotaceae* | 5 | 2.26 | 2 | 1.96 | 40.00 |
|  | *Endocladiaceae* | 2 | 0.90 | 2 | 1.96 | 100.00 |
|  | *Halymeniaceae* | 7 | 3.17 | 2 | 1.96 | 28.57 |
|  | *Laminariaceae* | 2 | 0.90 | 2 | 1.96 | 100.00 |
|  | *Poaceae* | 4 | 1.81 | 2 | 1.96 | 50.00 |
|  | *Scytosiphonaceae* | 4 | 1.81 | 2 | 1.96 | 50.00 |
|  | *Solieriaceae* | 5 | 2.26 | 2 | 1.96 | 40.00 |
|  | *Alariaceae* | 1 | 0.45 | 1 | 0.98 | 100.00 |
|  | *Apocynaceae* | 1 | 0.45 | 1 | 0.98 | 100.00 |
|  | *Asteraceae* | 3 | 1.36 | 1 | 0.98 | 33.33 |
|  | *Ceramiaceae* | 7 | 3.17 | 1 | 0.98 | 14.29 |
|  | *Chordaceae* | 1 | 0.45 | 1 | 0.98 | 100.00 |
|  | *Codiaceae* | 2 | 0.90 | 1 | 0.98 | 50.00 |
|  | *Desmarestiaceae* | 1 | 0.45 | 1 | 0.98 | 100.00 |
|  | *Droseraceae* | 1 | 0.45 | 1 | 0.98 | 100.00 |
|  | *Dryopteridaceae* | 1 | 0.45 | 1 | 0.98 | 100.00 |
|  | *Dunaliellaceae* | 1 | 0.45 | 1 | 0.98 | 100.00 |
|  | *Euphorbiaceae* | 1 | 0.45 | 1 | 0.98 | 100.00 |
|  | *Fabaceae* | 1 | 0.45 | 1 | 0.98 | 100.00 |
|  | *Gigartinaceae* | 2 | 0.90 | 1 | 0.98 | 50.00 |
|  | *Gloiosiphoniaceae* | 1 | 0.45 | 1 | 0.98 | 100.00 |
|  | *Hypneaceae* | 3 | 1.36 | 1 | 0.98 | 33.33 |
|  | *Iridaceae* | 1 | 0.45 | 1 | 0.98 | 100.00 |
|  | *Ishigeaceae* | 2 | 0.90 | 1 | 0.98 | 50.00 |
|  | *Juncaceae* | 1 | 0.45 | 1 | 0.98 | 100.00 |
|  | *Lamiaceae* | 2 | 0.90 | 1 | 0.98 | 50.00 |
|  | *Lecythidaceae* | 1 | 0.45 | 1 | 0.98 | 100.00 |
|  | *Lessoniaceae* | 1 | 0.45 | 1 | 0.98 | 100.00 |
|  | *Lythraceae* | 2 | 0.90 | 1 | 0.98 | 50.00 |
|  | *Meliaceae* | 1 | 0.45 | 1 | 0.98 | 100.00 |
|  | *Monostromataceae* | 2 | 0.90 | 1 | 0.98 | 50.00 |
|  | *Plumbaginaceae* | 2 | 0.90 | 1 | 0.98 | 50.00 |
|  | *Polygonaceae* | 2 | 0.90 | 1 | 0.98 | 50.00 |
|  | *Primulaceae* | 1 | 0.45 | 1 | 0.98 | 100.00 |
|  | *Rhodymeniaceae* | 1 | 0.45 | 1 | 0.98 | 100.00 |
|  | *Rubiaceae* | 2 | 0.90 | 1 | 0.98 | 50.00 |
|  | *Zosteraceae* | 1 | 0.45 | 1 | 0.98 | 100.00 |
|  | Other | 42 | 19.00 | 0 | 0.00 | 0.00 |
|  | Total | 221 | 100.00 | 102 | 100.00 | 46.15 |

a Percentage derived from division with the total number of species within *Viridiplantae* kingdom, *Eukaryota* superkingdom and *Bacteria* superkingdom (n=221).

b Percentage derived from division with the number of species with AA within *Viridiplantae* kingdom, *Eukaryota* superkingdom and *Bacteria* superkingdom (n=102).

c Percentage derived from division with the number of species within each corresponding family.

**Table S6.** The distribution of marine organisms with anticancer activity (AA) in different phylum of *Viridiplantae* kingdom, *Eukaryota* superkingdom and *Bacteria* superkingdom.

| **NO** | **Phylum** | **Number** | **Percentage^a^** | **No. of AA** | **Percentage^b^** | **Percentage^c^** |
| --- | --- | --- | --- | --- | --- | --- |
|  | *Streptophyta* | 67.00 | 30.32 | 35 | 34.31 | 52.24 |
|  | *Chlorophyta* | 20.00 | 9.05 | 8 | 7.84 | 40 |
|  | *Cyanobacteria* | 9.00 | 4.07 | 6 | 5.88 | 66.67 |
|  | *Streptophyt* | 2.00 | 0.9 | 2 | 1.96 | 100 |
|  | *Phaeophyceae* | 1.00 | 0.45 | 1 | 0.98 | 100 |
|  | *Bacillariophyta* | 8.00 | 3.62 | 0 | 0 | 0 |
|  | Other | 114.00 | 51.58 | 50 | 49.02 | 43.86 |
|  | Total | 221.00 | 100 | 102 | 100 | 46.15 |

a Percentage derived from division with the total number of species within *Viridiplantae* kingdom, *Eukaryota* superkingdom and *Bacteria* superkingdom (n=221).

b Percentage derived from division with the number of species with AA within *Viridiplantae* kingdom, *Eukaryota* superkingdom and *Bacteria* superkingdom (n=102).

c Percentage derived from division with the number of species within each corresponding phylum.

**Table S7.** The distribution of marine organisms with anticancer activity (AA) in different classes of *Viridiplantae* kingdom, *Eukaryota* superkingdom and *Bacteria* superkingdom.

| **NO** | **Class** | **Number** | **Percentage^a^** | **No. of AA** | **Percentage^b^** | **Percentage^c^** |
| --- | --- | --- | --- | --- | --- | --- |
|  | *Florideophyceae* | 66 | 29.86 | 24 | 23.53 | 36.36 |
|  | *Phaeophyceae* | 37 | 16.74 | 23 | 22.55 | 62.16 |
|  | *Ulvophyceae* | 19 | 8.6 | 7 | 6.86 | 36.84 |
|  | *Liliopsida* | 15 | 6.79 | 5 | 4.90 | 33.33 |
|  | *Bangiophyceae* | 10 | 4.52 | 3 | 2.94 | 30.00 |
|  | *Chlorophyceae* | 1 | 0.45 | 1 | 0.98 | 100.00 |
|  | *Bacillariophyceae* | 4 | 1.81 | 0 | 0.00 | 0.00 |
|  | *Fragilariophyceae* | 4 | 1.81 | 0 | 0.00 | 0.00 |
|  | Others | 65 | 29.41 | 39 | 38.24 | 60.00 |
|  | Total | 221 | 100 | 102 | 100.00 | 46.15 |

a Percentage derived from division with the total number of species within *Viridiplantae* kingdom, *Eukaryota* superkingdom and *Bacteria* superkingdom (n=221).

b Percentage derived from division with the number of species with AA within *Viridiplantae* kingdom, *Eukaryota* superkingdom and *Bacteria* superkingdom (n=102).

c Percentage derived from division with the number of species within each corresponding class.

**Table S8.** The distribution of marine organisms with anticancer activity (AA) in different order of of *Viridiplantae* kingdom, *Eukaryota* superkingdom and *Bacteria* superkingdom.

| **No.** | **Order** | **Number** | **Percentage^a^** | **No. of AA** | **Percentage^b^** | **Percentage^c^** |
| --- | --- | --- | --- | --- | --- | --- |
|  | *Fucales* | 20 | 9.05 | 13 | 0.00 | 0.00 |
|  | *Gigartinales* | 16 | 7.24 | 7 | 43.75 | 6.86 |
|  | *Malpighiales* | 8 | 3.62 | 7 | 87.50 | 6.86 |
|  | *Ceramiales* | 16 | 7.24 | 6 | 37.50 | 5.88 |
|  | *Oscillatoriales* | 9 | 4.07 | 6 | 66.67 | 5.88 |
|  | *Laminariales* | 5 | 2.26 | 5 | 100.00 | 4.90 |
|  | *Ulvales* | 11 | 4.98 | 4 | 36.36 | 3.92 |
|  | *Lamiales* | 5 | 2.26 | 4 | 80.00 | 3.92 |
|  | *Caryophyllales* | 15 | 6.79 | 3 | 20.00 | 2.94 |
|  | *Gracilariales* | 12 | 5.43 | 3 | 25.00 | 2.94 |
|  | *Bangiales* | 10 | 4.52 | 3 | 30.00 | 2.94 |
|  | *Poales* | 10 | 4.52 | 3 | 30.00 | 2.94 |
|  | *Gelidiales* | 8 | 3.62 | 3 | 37.50 | 2.94 |
|  | *Ectocarpales* | 7 | 3.17 | 3 | 42.86 | 2.94 |
|  | *Bryopsidales* | 6 | 2.71 | 3 | 50.00 | 2.94 |
|  | *Myrtales* | 4 | 1.81 | 3 | 75.00 | 2.94 |
|  | *Malvales* | 3 | 1.36 | 3 | 100.00 | 2.94 |
|  | *Halymeniales* | 7 | 3.17 | 2 | 28.57 | 1.96 |
|  | *Dictyotales* | 5 | 2.26 | 2 | 40.00 | 1.96 |
|  | *Corallinales* | 3 | 1.36 | 2 | 66.67 | 1.96 |
|  | *Ericales* | 3 | 1.36 | 2 | 66.67 | 1.96 |
|  | *Gentianales* | 3 | 1.36 | 2 | 66.67 | 1.96 |
|  | *Apiales* | 2 | 0.90 | 2 | 100.00 | 1.96 |
|  | *Solanales* | 2 | 0.90 | 2 | 100.00 | 1.96 |
|  | *Alismatales* | 2 | 0.90 | 1 | 50.00 | 0.98 |
|  | *Asterales* | 2 | 0.90 | 1 | 50.00 | 0.98 |
|  | *Sapindales* | 2 | 0.90 | 1 | 50.00 | 0.98 |
|  | *Asparagales* | 1 | 0.45 | 1 | 100.00 | 0.98 |
|  | *Chlamydomonadales* | 1 | 0.45 | 1 | 100.00 | 0.98 |
|  | *Desmarestiales* | 1 | 0.45 | 1 | 100.00 | 0.98 |
|  | *Fabales* | 1 | 0.45 | 1 | 100.00 | 0.98 |
|  | *Polypodiales* | 1 | 0.45 | 1 | 100.00 | 0.98 |
|  | *Rhodymeniales* | 1 | 0.45 | 1 | 100.00 | 0.98 |
|  | *Bacillariales* | 4 | 1.81 |  | 0.00 | 0.00 |
|  | *Fragilariales* | 3 | 1.36 |  | 0.00 | 0.00 |
|  | *Nemaliales* | 2 | 0.90 |  | 0.00 | 0.00 |
|  | *Asterale* | 1 | 0.45 |  | 0.00 | 0.00 |
|  | *Bonnemaisoniales* | 1 | 0.45 |  | 0.00 | 0.00 |
|  | *Cladophorales* | 1 | 0.45 |  | 0.00 | 0.00 |
|  | *Commelinales* | 1 | 0.45 |  | 0.00 | 0.00 |
|  | *Dipsacales* | 1 | 0.45 |  | 0.00 | 0.00 |
|  | *Fagales* | 1 | 0.45 |  | 0.00 | 0.00 |
|  | *Pandanales* | 1 | 0.45 |  | 0.00 | 0.00 |
|  | *Plocamiales* | 1 | 0.45 |  | 0.00 | 0.00 |
|  | *Ulotrichales* | 1 | 0.45 |  | 0.00 | 0.00 |
|  | Other | 1 | 0.45 |  | 0.00 | 0.00 |
|  | Total | 221 | 100.00 | 102 | 46.15 | 100.00 |

a Percentage derived from division with the total number of species within *Viridiplantae* kingdom, *Eukaryota* superkingdom and *Bacteria* superkingdom (n=221).

b Percentage derived from division with the number of species with AA within *Viridiplantae* kingdom, *Eukaryota* superkingdom and *Bacteria* superkingdom (n=102).

c Percentage derived from division with the number of species within each corresponding order.
